# Carbon Quantum Dots Derived from Different Carbon Sources for Antibacterial Applications

**DOI:** 10.3390/antibiotics10060623

**Published:** 2021-05-24

**Authors:** Yanyan Wu, Cong Li, Henny C. van der Mei, Henk J. Busscher, Yijin Ren

**Affiliations:** 1 University of Groningen and University Medical Center of Groningen, Department of Orthodontics, Hanzeplein 1, 9700 RB Groningen, The Netherlands; y.wu@umcg.nl (Y.W.); y.ren@umcg.nl (Y.R.); 2 College of Chemistry, Chemical Engineering and Materials Science, Soochow University, 199 Ren’ai Rd, Suzhou 215123, China; licong_0213@163.com; 3 University of Groningen and University Medical Center Groningen, Department of Biomedical Engineering, Antonius Deusinglaan 1, 9713 AV Groningen, The Netherlands; h.c.van.der.mei@umcg.nl

**Keywords:** nanoparticles, precursor, reactive oxygen species, photodynamic activity, biofilm, infection, size control, dispersal, antibiotics, synergy

## Abstract

Nanoparticles possess unique features due to their small size and can be composed of different surface chemistries. Carbon quantum dots possess several unique physico-chemical and antibacterial activities. This review provides an overview of different methods to prepare carbon quantum dots from different carbon sources in order to provide guidelines for choosing methods and carbon sources that yield carbon quantum dots with optimal antibacterial efficacy. Antibacterial activities of carbon quantum dots predominantly involve cell wall damage and disruption of the matrix of infectious biofilms through reactive oxygen species (ROS) generation to cause dispersal of infecting pathogens that enhance their susceptibility to antibiotics. Quaternized carbon quantum dots from organic carbon sources have been found to be equally efficacious for controlling wound infection and pneumonia in rodents as antibiotics. Carbon quantum dots derived through heating of natural carbon sources can inherit properties that resemble those of the carbon sources they are derived from. This makes antibiotics, medicinal herbs and plants or probiotic bacteria ideal sources for the synthesis of antibacterial carbon quantum dots. Importantly, carbon quantum dots have been suggested to yield a lower chance of inducing bacterial resistance than antibiotics, making carbon quantum dots attractive for large scale clinical use.

## 1. Introduction

The spread of antibacterial-resistant infections is considered to be one of the largest threats to public health [1]. Concerns particularly arise from prolonged use and overuse of antibiotics, both in clinical and in agricultural practices [2,3]. Since bacteria can adapt quickly to many hostile environmental conditions, including antibiotic exposure, antibiotic resistance is rampant [4], and occurring faster and faster after the market introduction of a new antibiotic. Clinically, antibiotic-resistant bacterial infections are highly challenging to treat, often resulting in morbidity and mortality [5]. New, innovative antibacterial agents on a non-antibiotic basis, or that can bypass bacterial mechanisms of developing antibiotic-resistance [6], are therefore urgently needed. 

Nanoparticles possess many unique features due to their extremely small size. Nanoparticles can be composed of highly different surface chemistries and generally range in size from several nanometers to hundreds of nanometers (for comparison, a gold atom has a diameter of one third of a nanometer), yielding large surface areas and highly diverse shapes. These unique features can also provide nanoparticles with antibacterial activity. 

Carbon quantum dots have a diameter of less than 10 nm. Carbon quantum dots can be classified according to their structure and composition. Graphene dots consist of a single or a few graphene layers [7,8], and polymer dots are aggregated or cross-linked polymer structures around a hollow or carbon core [9,10,11]. Carbon nanodots are carbon-based quantum dots [12,13]. All carbon quantum dots possess sp^2^ or sp^3^-hybridized carbon domains that provide stability and special optical features, including giant Stokes shifts in photoluminescence and a strong dependence of emission color on excitation wavelength, depending on their size, core structure and composition [14]. Carbon quantum dots are widely investigated for possible application in bio-imaging, drug delivery, biosensors, cancer therapy and antibacterial applications [15,16,17,18,19]. 

Carbon quantum dots can be obtained through a variety of methods (Table 1). Size control is critical in the synthesis of carbon quantum dots [20] and, collectively, it can be seen from Table 1 that poor size control is the main problem in the synthesis of carbon quantum dots. Accordingly, synthesis of carbon quantum dots is often followed by dialysis [21], centrifugation [22] or filtration [23] to obtain a uniform size distribution.

“Top-down” synthesis methods break down large carbon-rich materials as a carbon source, whereas “bottom-up” methods synthesize carbon quantum dots from small precursor molecules. Both the method applied as well as the carbon source or precursor used are determinant factors for the final physico-chemical and functional properties of carbon quantum dots, including their biocompatibility and antibacterial efficacy [71,72]. Therefore, we first provide a more detailed description of the different synthesis methods listed in Table 1, and a summary of physico-chemical and functional properties, including antibacterial activity of carbon quantum dots made using different methods and carbon sources, with the aim of providing guidelines for choosing methods and carbon sources that yield optimal antibacterial activity of carbon quantum dots.

## 2. Methods for Synthesizing Carbon Quantum Dots

In the forthcoming sections, we will describe the different methods for synthesizing carbon quantum dots listed in Table 1 in more detail. 

### 2.1. Acidic Oxidation

**Figure 1:**
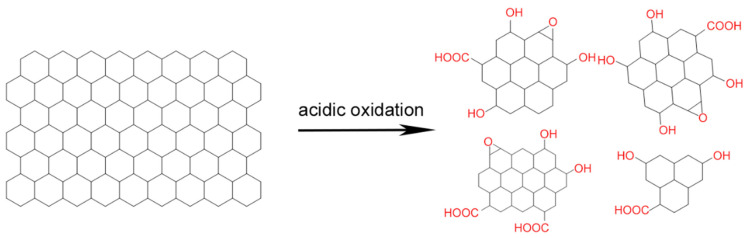
Acidic oxidation of a carbon source, yielding carbon quantum dots with different oxygen-containing groups.

**Figure 2:**
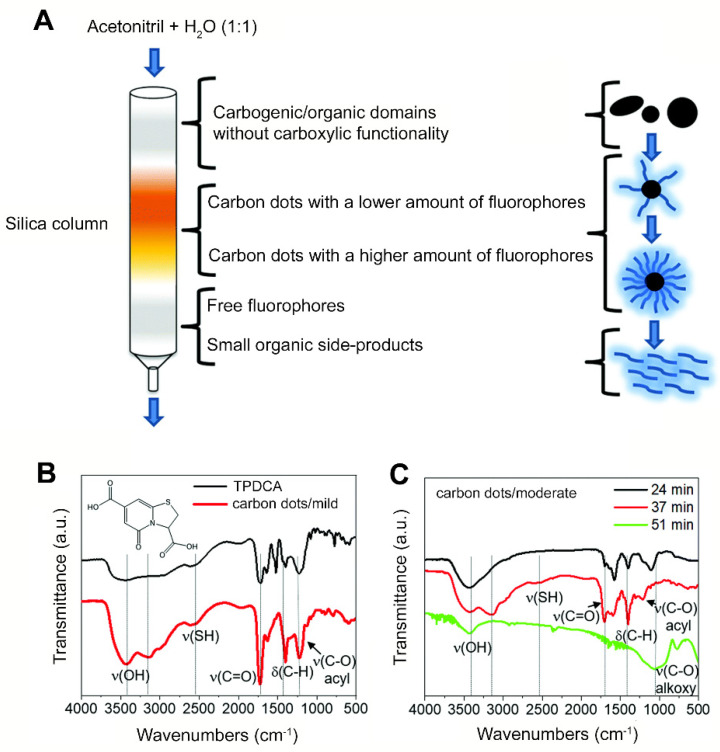
Purification of carbon quantum dots, obtained by oxidation of cysteine using citric acid under hydrothermal conditions, via column chromatography: (**A**) Different fractions obtained in the purification of carbon quantum dots by chromatography. (**B**) FTIR spectra of dried free-floating fluorophores (TPDCA) first leaving the chromatography column and carbon quantum dots synthesized during acidification for 3 h at 150 °C (mild acidification). (**C**) FTIR spectra of different fractions of carbon quantum dots synthesized during acidification for 6 h at 200 °C (moderate acidification), obtained after different retention times in the chromatography column. Note the disappearance of the absorption bands due to free-floating fluorophores. Reproduced with permission from Ref. [45], copyright Royal Society of Chemistry, 2019.

Acidic oxidation is a relatively simple method in which mainly inorganic carbon sources are oxidized by exposure to HNO_3_, H_2_SO_4_, NaNO_3_, KMnO_4_ or other oxidizers at temperatures up to 140 °C. Reaction times to break down a carbon source into carbon quantum dots range from 12 to 48 h [21,24,25,26,27,28,29,30,31,32,33,34,35,36]. The acidic conditions combined with high temperatures necessitate extreme care when applying acidic oxidation to synthesize carbon quantum dots. Different carbon sources, such as coal [25,27], fullerene C_60_ [26], carbon nano-powders [24,29,30,34], carbon fibers [35], candle soot [21] and graphite [31,32,33] can be applied for acidic oxidation, resulting in carbon quantum dots. Typically, cleavage occurs at carbon-carbon bonds, introducing negatively charged, oxygen-containing groups, such as C-O, C=O or O-H, onto the surface of the resultant carbon quantum dots (Figure 1). Thus prepared, carbon quantum dots are hydrophilic and highly suitable for further chemical modification [25,26]. In order to obtain a uniform size distribution and remove acid residues and molecular intermediates, carbon quantum dots synthesized by acidic oxidation usually need purification (Figure 2) [45].

### 2.2. Pyrolysis

Pyrolysis can be applied to synthesize carbon quantum dots by heating carbon sources to reaction temperatures up to 1500 °C [42]. Pyrolysis allows one to synthesize carbon quantum dots within several hours, but with a highly variable yield ranging from 0.01% [37] to 51% [19], depending on the carbon source and treatment conditions applied. Pyrolysis can typically use widely different carbon sources, ranging from silicon carbide [42], flour [43], gentamicin sulfate [39], ammonium citrate [19] or dopamine [38], together with spermidine, citric acid or dicyandiamide [41]. In pyrolysis, carbon quantum dots result from intermolecular dehydration, carbonization and condensation, and the resulting carbon skeleton is held together by chemical groups such as –SO_2_, –HSO_3_ and –H_2_PO_4_ [44]. 

### 2.3. Hydrothermal Synthesis

**Figure 3:**
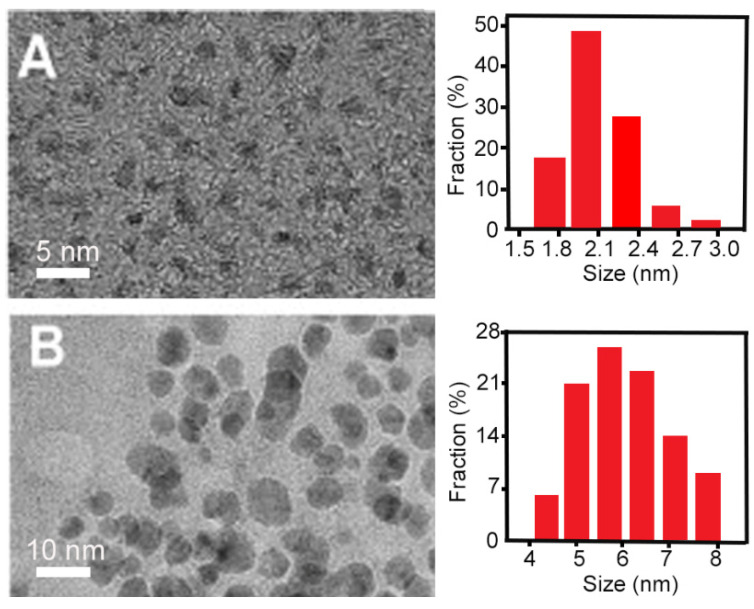
Dialysis of carbon quantum dots hydrothermally synthesized using graphite powders to obtain quantum dots with a uniform size distribution: (**A**) TEM micrographs and size distribution of carbon quantum dots that passed through a dialysis bag, showing smaller particles with a uniform size distribution. (**B**) TEM micrographs and size distribution of carbon quantum dots that did not pass through a dialysis bag, showing larger particles with a wide size distribution. Reproduced with permission from Ref. [52], copyright Elsevier, 2014.

Hydrothermal synthesis can either be applied as a top-down or bottom-up method to prepare carbon quantum dots, using natural biomass, graphite, polymers or small organic molecules as carbon sources. In hydrothermal synthesis, carbon sources are heated in an aqueous suspension to reaction temperatures ranging from 120 °C to 260 °C. Hydrothermal synthesis of carbon quantum dots is commonly performed in an autoclave, requiring between 4 h and 24 h [22,23,46,47,48,49,50,51,53]. Hydrothermally obtained carbon quantum dots do not possess a uniform size (Figure 3) and require dialysis or filtration to obtain a uniform size distribution.

### 2.4. Microwave-Assisted Synthesis

Microwave-assisted synthesis is applied in combination with acidic oxidation or hydrothermal synthesis methods as a convenient way to achieve the desired high-reaction temperatures [21,54,55,56,57]. Compared with conventional heating systems, microwave-heating is more targeted at the carbon source, which shortens the reaction times from several days [29] to hours [57] or even minutes [21]. 

### 2.5. Laser Irradiation

Laser irradiation can be a top-down or bottom-up method to synthesize carbon quantum dots by pulsed, high energy laser irradiation of carbon sources, such as graphite [59,73], benzene [58] or toluene [60], reducing the time required for synthesis to several hours or even minutes. Carbon quantum dots derived using laser ablation usually need centrifugation or filtration to remove large nanoparticles.

### 2.6. Electrochemical Synthesis

**Figure 4:**
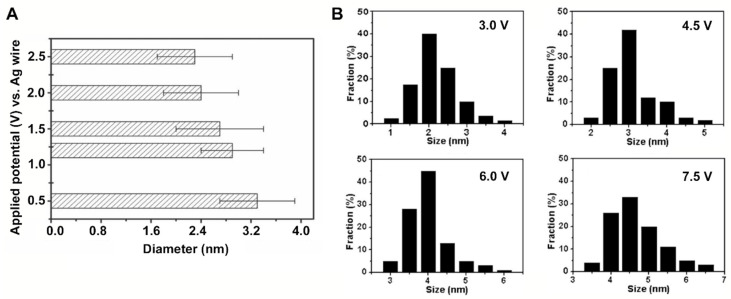
Size control in the electrochemical synthesis of carbon quantum dots: (**A**) Diameters of carbon quantum dots electrochemically top-down exfoliated from carbon sheets, applying different voltages. Reproduced with permission from Ref. [63], copyright Wiley, 2011. (**B**) Diameters of carbon quantum dots electrochemically obtained in a bottom-up approach from ethanol, applying different voltages. Reproduced with permission from Ref. [66], copyright Wiley, 2014.

In electrochemical synthesis of carbon quantum dots, a counter electrode and a working electrode made of an appropriate carbon source, such as graphite rods [12,64,65], carbon fibers [63] or multi-walled carbon nanotubes [62], are used. As an electrolyte solution, a degassed solution of acetonitrile supplemented with 0.1 M tetrabutylammonium [62], water [64] or phosphate solutions [65] has been applied. Upon application of a voltage across the electrodes, carbon quantum dots can be top-down exfoliated from the carbon source constituting the working electrode. As a major advantage, the size of carbon quantum dots can be precisely controlled by adjusting the electrode potential and current density (see Figure 4A). 

Electrochemical synthesis can also be employed bottom-up using alcohols or vitamin C as precursor molecules to synthesize carbon quantum dots under constant voltage conditions through molecular crosslinking and dehydration [61,66]. A higher applied potential resulted in larger carbon quantum dots (Figure 4B), which is in contrast to using bulk carbon materials, as the small molecules would undergo crosslinking and dehydration to form carbon quantum dots. 

### 2.7. Nanoreactor-Assisted Synthesis

**Figure 5:**
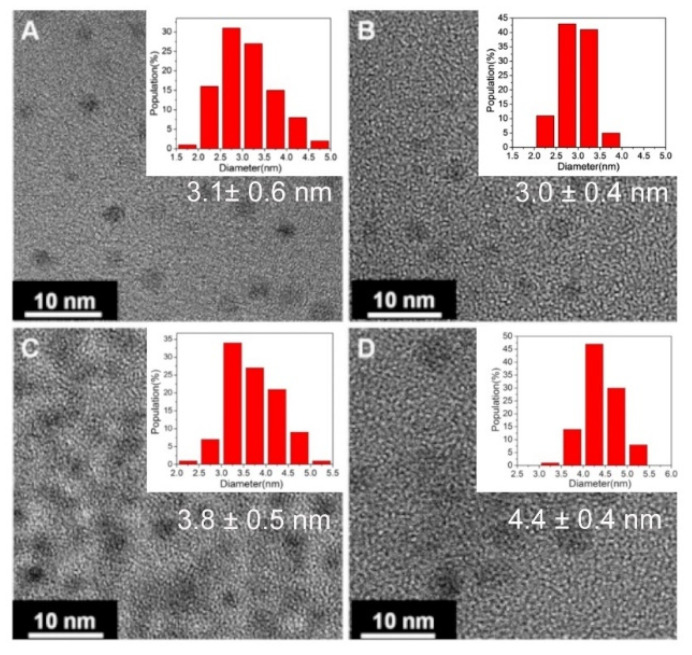
TEM micrographs of carbon quantum dots prepared from different organic precursors in a soft–hard template nanoreactor consisting of Pluronic P123 micelles and mesoporous silica. Carbon quantum dots were synthesized from: (**A**) 1,3,5-trimethylbenzene, (**B**) diaminebezene, (**C**) pyrene and (**D**) phenanthroline. The insets show the size distributions. Reproduced with permission from Ref. [69], copyright Royal Society of Chemistry, 2013.

Size control is one of the major challenges in the synthesis of carbon quantum dots. This has stimulated the development of templates for the synthesis of carbon quantum dots in a confined volume (“nanoreactors”). Nanoreactors are mostly mesoporous silica nanoparticles because they possess high thermal stability, uniform pore size distribution and large pore volume [67,68,69,70]. Nanoreactor-assisted synthesis is usually performed through bottom-up pyrolysis, after the absorption of precursor molecules which are dissolved in a fluid phase into the nanoreactor. The release of the synthesized carbon dots can be achieved, e.g., by exposure of the nanoreactor to alkaline solutions, necessitating dialysis to purify the resulting carbon quantum dot suspension. Nanoreactor construction can be difficult and time-consuming to prepare, but the use of nanoreactors directly yields highly monodisperse carbon quantum dots with narrow size distribution (Figure 5).

## 3. Physico-Chemical and Functional Properties of Carbon Quantum Dots

Carbon quantum dots have physico-chemical and functional properties, including antibacterial activity, that depend on the carbon source or precursor molecules used. In this section, we will briefly overview physico-chemical and functional properties characteristic to carbon quantum dots derived from different sources, including the coating of carbon nanoparticles to enhance their functionality. In Section 4, we will deal with the antibacterial activities of carbon quantum dots.

### 3.1. Carbon Quantum Dots Derived from Organic Carbon Sources

**Figure 6:**
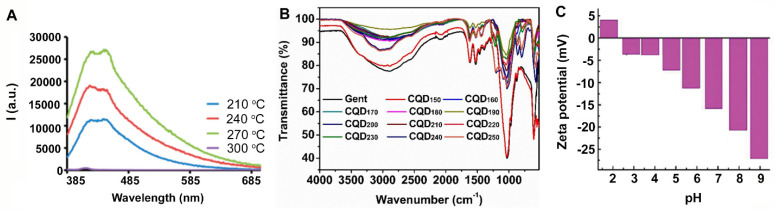
Physico-chemical properties of carbon quantum dots prepared from organic reagents: (**A**) Fluorescence spectra of carbon quantum dots pyrolytically synthesized from spermidine at different temperatures. Reproduced with permission from Ref. [37], copyright American Chemical Society, 2017. (**B**) Similarities in infrared absorption spectra between gentamicin sulfate and carbon quantum dots (CQD_T_) derived through calcination at different temperatures (T), demonstrating preservation of the active structure of gentamicin sulfate. Reproduced with permission from Ref. [39], copyright Royal Society of Chemistry, 2020. (**C**) Zeta potentials at different pH of carbon quantum dots hydrothermally synthesized from tri-basic citric acid and dicyandiamide. Reproduced with permission from Ref. [77], copyright Royal Society of Chemistry, 2014.

Organic reagents can be employed in various methods for the preparation of carbon quantum dots (see Table 1), and range from polyamine [19,21,37,38], quaternary ammonium salt [47,74], gentamicin [39], poly(sodium-4-styrene sulfonate) [46], polyvinylpyrrolidone [46], metronidazole [49] citric acid and polyethyleneimine [75], vitamin C [61] to benzene [58], polyoxyethylene−polyoxypropylene−polyoxyethylene Pluronic 68 and phosphoric acid [76]. The quantum yield of carbon dots prepared from organic sources using heating-based carbonization increases with increasing reaction temperature (Figure 6A) [37]. However, when carbonization temperatures become too high, carbon quantum dots obtained can become difficult to suspend [37,38,39]. 

Carbon quantum dots can bear similarity to the organic reagents that they are derived from (Figure 6B) [19,21,37,38,39,48]. Similarities disappear, however, when reaction temperatures become too high. Accordingly, carbon quantum dots derived from amide- and amine-rich sources have a more positive surface charge than carbon dots derived from acids and sulfonate groups [19,21,37,38,46,49,77]. Zeta potentials of carbon quantum dots synthesized from tri-basic citric acid and dicyandiamide under hydrothermal conditions were pH dependent. Below the lowest pKa of citric acid (2.94), citric acid-derived carbon quantum dots were positively charged due to protonation (Figure 6C) while, above pH 3 and its two higher pKa values (4.28 and 5.21), carbon quantum dots became negatively charged [77]. 

### 3.2. Carbon Quantum Dots Derived from Inorganic Carbon Sources 

Carbon nanopowders and graphite are the most common inorganic carbon sources used for the synthesis of carbon quantum dots. Inorganic carbon sources can be applied in a wide variety of synthesis methods (Table 1). 

Graphite has been employed as a carbon source using acidic oxidation to yield carbon quantum dots [31,32,33] bearing a negative charge with zeta potentials around −22 mV due to the oxygen-containing groups on the surface. Quantum yields ranged from 12.5% to 33% depending on the nitrogen content. When the reaction was performed in the presence of ammonia, nitrogen was introduced in carbon quantum dots. These nitrogen-doped carbon quantum dots exhibited higher quantum yields than nitrogen free carbon quantum dots, and were able to generate a higher amount of reactive oxygen species (ROS) under photoexcitation [32]. Additionally, carbon quantum dots prepared from graphite have been reported to possess peroxidase-like activity, which catalyzed H_2_O_2_ decomposition and generate hydroxyl radicals [54].

### 3.3. Carbon Dots Derived from Natural Carbon Sources

**Figure 7:**
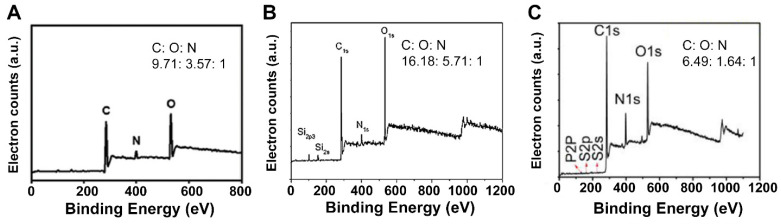
XPS spectra of carbon quantum dots prepared from natural carbon sources: (**A**) XPS spectra of carbon quantum dots derived from *Artemisia argyi* leaves. Reproduced with permission from Ref. [78], copyright Royal Society of Chemistry, 2020. (**B**) XPS spectra of carbon quantum dots derived from flour. Reproduced with permission from Ref. [83], copyright Elsevier. (**C**) XPS spectra of carbon quantum dots derived from *Lactobacillus plantarum* bacteria. Ratios presented are between at% of the elements indicated. Reproduced with permission from ref. [23], copyright Frontiers, 2018.

Compared to synthetic carbon sources, natural carbon sources are ecologically friendly, cost-effective and easy to obtain [23,78,79,80,81]. Natural carbon sources such as leaves [78], paper [81], honey [82], flour [83] and bacteria [23] have all been applied to synthesize carbon quantum dots. Natural carbon sources are mostly applied in pyrolysis, hydrothermal heating and microwave-assisted methods to synthesize carbon quantum dots (see Table 1). Carbon quantum dots prepared from highly different natural carbon sources roughly possess comparable elemental compositions. However, natural carbon sources often contain non-carbon atoms that have replaced a carbon atom in the molecular structure of the molecules. Typically, the presence of such hetero-atoms, including, e.g., nitrogen, phosphorus or sulphur, can provide special properties to carbon quantum dots during synthesis, without additional surface coating [32,33,77]. Using *Artemisia argyi* leaves as the carbon precursor, carbon quantum dots were synthesized simulating smoking of the leaves [78] that consisted mainly of carbon, oxygen and nitrogen (Figure 7A). Carbon quantum dots derived from cigarette smoke, i.e., tobacco leaves, had a similar composition as *Artemisia argyi* leave-derived carbon quantum dots [79]. Carbon quantum dots prepared from flour under microwave-assist had a quantum yield of 5% and were mainly composed of carbon, oxygen and nitrogen (Figure 7B), resulting in a slightly negative zeta potential of −4 mV [83]. Carbon quantum dots hydrothermally derived from the bacterium *Lactobacillus plantarum* at a quantum yield of 10% were also mainly composed of carbon, with oxygen and nitrogen as the main hetero-atoms, next to small amounts of phosphorus and sulphur (Figure 7C). These hetero-atoms yielded highly negatively zeta potentials of −22 mV. Thus, it can be concluded that the similarity between carbon quantum dots and their natural carbon sources is mainly due to the presence of hetero-atoms. Differences in the prevalence of hetero-atoms are responsible for the different properties of carbon quantum dots derived from natural sources. 

### 3.4. Surface Modification of Carbon Quantum Dots to Enhance their Functionality 

Carbon quantum dots in the absence of surface modification can be weakly fluorescent, or do not exhibit the functionality desired. Accordingly, carbon quantum dots can be surface modified with organic molecules to generate strong fluorescence, photodynamic effects and functionalities that include antibacterial activity. The photodynamic effect of carbon quantum dots synthesized from carbon nanopowders by acidic oxidation could be enhanced by amidation with 2,2′-(ethylenedioxy) bis(ethylamine), yielding positively charged carbon quantum dots with a quantum yield between 7 and 27%, depending on the effectiveness of surface passivation [24,29,30,34]. Carbon quantum dots hydrothermally prepared from citric acid and polyethyleneimine were positively charged at pH 5.0 due to the possession of cationic amino groups, and could be made negatively charged by modification with 2,3-dimethylmaleic-anhydride [75]. 

## 4. Antibacterial Activities of Carbon Quantum Dots

Antibacterial activity is often misused as an expression, as it can relate to several different mechanisms [19,21,46,47,54,61,74]. The mildest antibacterial activity relates to growth inhibition, impeding multiplication of bacteria and allowing the host immune system ample time to deal with infecting bacteria [23]. Killing is the strongest expression of antibacterial activity and implies that a bacterium has permanently lost its ability to be metabolically active and multiply [19,21,37,38,46,58,61,76]. Both growth inhibition and killing can be preceded or accompanied by cell wall damage [39,47,78,84]. 

### 4.1. Bacterial Killing by Carbon Quantum Dots 

Direct antibacterial activity of carbon quantum dots is due in a major part to oxidative stress induced by ROS [85] generated by carbon quantum dots. In low concentrations, ROS acts as a signaling molecule within cells in response to, e.g., a pathogen challenge. Oxidative stress develops when the level of ROS generation exceeds the natural antioxidant defense of a bacterium [86] and, when overly present, it causes oxidative damage to nucleotides, lipids and proteins, leading to cell wall damage and bacterial death [21,46]. Hetero-atoms in carbon quantum dots enhance the generation of ROS due to extra free electron incorporation in carbon dots [46]. The lifetime of ROS is generally short, depending on the type of ROS. As compared with other types of ROS, hydrogen peroxide has the longest lifetime of about 1 ms. Other types of ROS have lifetimes in the µs-range [87]. As a result, ROS can only diffuse over short distances up to several 100 nm, allowing diffusion across lipid membranes. Nevertheless, these short lifetimes of ROS necessitate generation in the close vicinity of its target pathogens for effective antibacterial activity. Nitrogen hetero-atoms in carbon quantum dots will yield positively charged groups and enhance the electrostatic double-layer attraction to negatively charged bacterial cell surface [46] that will aid ROS generation close to target pathogens. Additionally, a positive charge on its own, i.e., without ROS generation can cause antibacterial effects. 

Positively charged carbon quantum dots prepared from spermidine [19,37] or quaternary ammonium salts [47] have been demonstrated to adhere strongly to proteins, porins and bacterial cell wall peptidoglycan, resulting in inhibition of cell wall synthesis in Gram-positive and Gram-negative bacteria, persister cells and antimicrobial-resistant bacteria. As a result, the minimal inhibitory concentrations (MICs) of carbon quantum dots prepared from spermidine [19] or quaternary ammonium salts [47] for different Gram-positive and Gram-negative pathogens were up to 250,000-fold lower than those of spermidine (around 26 mg/mL), and up to 8-fold lower than those of ammonium salts (around 16 µg/mL [88]), indicating that their carbonization enhances antibacterial activity (also see Table 2). The MICs of these carbon quantum dots in terms of weight per unit volume are therewith considerably lower than for antibiotics (MIC of MRSA for gentamicin, rifampicin, penicillin and methicillin was 8, 8, >64 and >64 μg/mL, respectively, and the MIC for ampicillin-resistant *E. coli* for gentamicin, rifampicin, penicillin and methicillin was >64, 4, >64, >64 μg/mL, respectively [47]). Carbon quantum dots derived from *Artemisia argyi* leaves killed only Gram-negative bacteria by inhibiting enzyme activity exclusively related with Gram-negative bacterial cell wall synthesis [78]. After damaging the cell wall, carbon quantum dots gain access to the interior of a bacterium to cause oxidative damage to its DNA [21].

The inheritance of antibacterially active chemical functionalities by carbon quantum dots as derived, e.g., from carbonization of gentamicin sulfate (Figure 6B) forms another mechanism of antibacterial activity by carbon quantum dots [39]. Based on a weight comparison, MICs of gentamicin sulfate-derived carbon quantum dots by carbonization at low temperature (150 °C) were roughly of the same order of magnitude as the MIC of gentamicin against *S. aureus* (0.18 µg/mL) or *E. coli* (3 µg/mL). Carbonization at temperatures above 190–200 °C caused loss of antibacterial activity. However, the MICs of gentamicin-derived carbon quantum dots when carbonized at 180 °C were lower at an acidic biofilm pH (see Table 2) those of gentamicin for *S. aureus* (0.2 µg/mL) and *E. coli* (23 µg/mL) [39]. 

### 4.2. Carbon Quantum Dots as a Biofilm Dispersant

In infection, bacteria not only adhere to each other to form aggregates, but also to mammalian cell surfaces, bone or tooth structures or prosthetic implant surfaces. Once it has adhered, a bacterium adapts to its substratum surface and starts producing a protective matrix composed of extracellular polymeric substances (EPS) that enwraps them into a biofilm [94,95]. The biofilm-mode of growth protects the inhabiting bacteria against the host immune system and antibiotic penetration [95,96]. Accordingly, disruption of the EPS matrix may also be considered as a mechanism of antibacterial activity, as it causes detachment of bacteria into a planktonic state (“biofilm dispersal”) that makes them amenable to the host’s immune system and antibiotics [74,75,94]. Interestingly, dimethyloctadecyl-[3-(trimethoxysilyl)propyl] ammonium chloride-derived carbon quantum dots not only dispersed *S. aureus* biofilms but also killed biofilm inhabitants [74], but this occurred at comparatively high carbon quantum dot concentrations of around 1000 μg/mL. Citric acid/polyethyleneimine-derived carbon dots modified with 2,3-dimethylmaleic anhydride exclusively dispersed biofilm of non-EPS producing *S. epidermidis* at concentrations of 125 μg/mL, but did not kill staphylococci up to at least 1000 μg/mL [75].

### 4.3. Carbon Quantum Dots and Induction of Resistance

**Figure 8:**
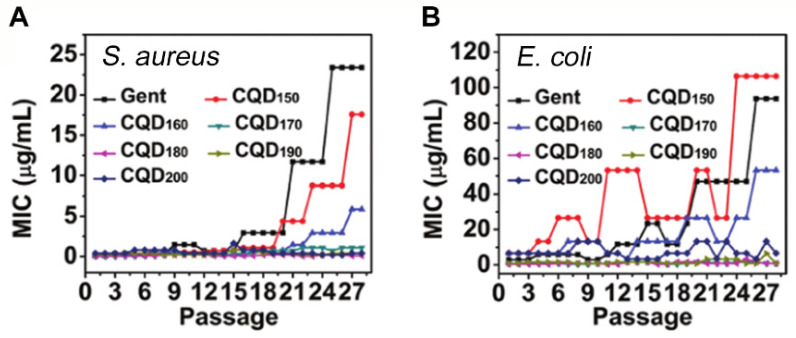
Minimal Inhibitory Concentrations (MIC) as a function of the number of times bacteria have been serially passaged in presence of increasing concentrations of gentamicin or gentamicin-derived carbon quantum dots. Carbon quantum dots synthesized by calcination at different temperatures, T (sub-script), from gentamicin (GENT), induce less resistance of (**A**) a Gram-positive *S. aureus* and (**B**) a Gram-negative *E. coli* strain than gentamicin used as a carbon source. Reproduced with permission from Ref. [39], copyright Royal Society of Chemistry 2020.

In addition to antibacterial efficacies summarized for carbon quantum dots in Table 2, it is important to notice that carbon quantum dots not only show good antibacterial activity but also possess a reduced risk for the development of antibiotic resistance compared to antibiotics (Figure 8) [39]. 

Nitrogen-doped carbon quantum dots, hydrothermally synthesized from a bis-quaternary ammonium salt, for instance, exhibited lower MIC than many common antibiotics, while inducing less resistance within an MRSA strain than penicillin [47]. Reduced risk of antimicrobial resistance might arguably be the most promising feature of carbon quantum dots, as many new antimicrobials lose efficacy within shorter and shorter periods of time [97], making the market introduction and clinical application of new antibiotics unlikely [98]. Carbon quantum dots probably evade mechanisms of bacterial resistance development by bacterial membrane disruption and ROS generation [46], instead of targeting a specific stage in the metabolic pathway of bacteria [39]. On the downside of this, bacteria have been shown to upregulate their antioxidant defense mechanism for scavenging ROS causing oxidative stress [99]. Whether or not this is a prelude towards bacterial resistance to ROS remains to be seen.

### 4.4. Mechanisms of Antibacterial Activity of Carbon Quantum Dots

**Figure 9:**
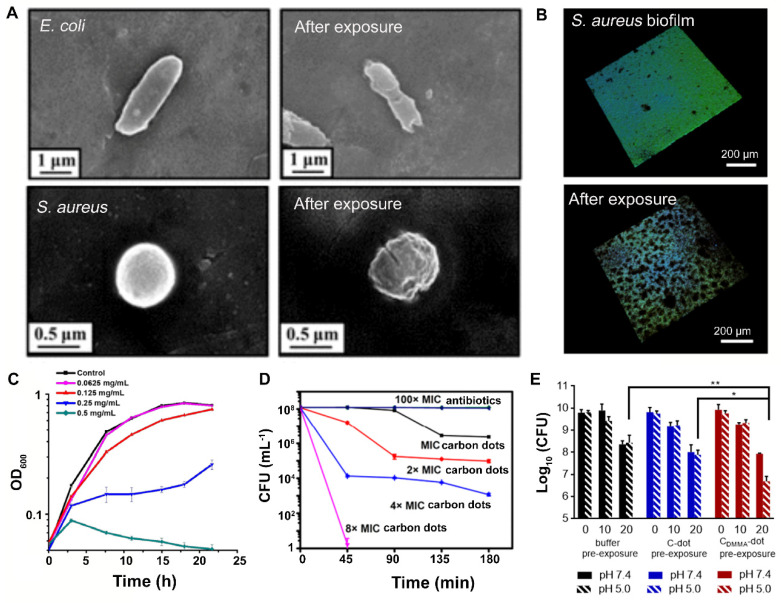
Examples of the antibacterial activity of carbon quantum dots according to different antibacterial mechanisms and synergistic use with antibiotics: (**A**) Cell wall damage to *E. coli* and *S. aureus*, inflicted upon exposure to cationic carbon quantum dots, pyrolytically synthesized from spermidine. Reproduced with permission from Ref. [37], copyright American Chemical Society, 2017. (**B**) Disruption of the EPS matrix of a *S. aureus* biofilm by carbon quantum dots synthesized from carbon fibers by acidic oxidation, causing biofilm dispersal. Reproduced with permission from Ref. [94], copyright American Chemical Society, 2019. (**C**) Growth inhibition of *P. aeruginosa* upon exposure to different concentrations of carbon quantum dots, hydrothermally synthesized from aminoguanidine and citric acid. Reproduced with permission from Ref. [84], copyright American Chemical Society, 2019. (**D**) Killing of stationary-phase MRSA upon exposure to nitrogen-doped carbon quantum dots, hydrothermally synthesized from a bis-quaternary ammonium salt, as compared with killing achieved by penicillin and gentamicin. MRSA were exposed to different concentrations of carbon quantum dots. Reproduced with permission from Ref. [47], copyright American Chemical Society, 2019. (**E**) Pre-exposure of *S. epidermidis* ATCC12228 biofilms to carbon quantum dots without (C-dots) and with 2,3-dimethylmaleic-anhydride (DMMA) functionalization (C_DMMA_-dots) yielded enhanced killing upon 72 h exposure to vancomycin at pH 5, as occurring in infectious biofilms. * *p* ˂ 0.05 and ** *p* ˂ 0.01 indicate significant differences with respect to vancomycin in absence of prior carbon dot exposure or prior exposure to C-dots (one way ANOVA). Reproduced with permission from Ref. [75], copyright ELSEVIER, 2020.

The summary of antibacterial activities of carbon quantum dots presented in Table 2 and the above discussion of their mechanism lead to the conclusion that the antibacterial mechanisms of carbon quantum dots mainly comprise cell wall damage (Figure 9A) [37] and disruption of the EPS matrix, causing biofilm dispersal (Figure 9B) [94]. Growth inhibition (Figure 9C) [84] and killing (Figure 9D) [47] were reported as possible mechanisms of antibacterial activity of carbon quantum dots in a significantly smaller number of papers. Little is known about the molecular mechanism of antibacterial activity once carbon quantum dots have gained intra-cellular access through cell wall damage. It is likely that carbon quantum dots affect gene expression [90].

### 4.5. Gram-Positive vs. Gram-Negative Strains

The different mechanisms through which carbon quantum dots exert antibacterial activities all act across both Gram-positive and Gram-negative bacterial strains. However, antibacterial efficacies of carbon quantum dots have been suggested to be slightly stronger for Gram-positive than for Gram-negative bacterial strains due to the possession of an inner and outer lipid membrane by Gram-negative bacteria consisting of lipids, proteins and lipopolysaccharides that make intracellular entry of carbon quantum dots more difficult [100]. This suggestion is confirmed by the generally higher MIC values of different quantum carbon dots against Gram-negative strains in Table 2. Table 2 furthermore shows that quite a number of carbon quantum dots from different sources selectively kill Gram-positive bacterial strains by causing cell wall damage [74,89] or Gram-negative ones [78,84]. Carbon quantum dots synthesized from aminoguanidine and citric acid have been described to highly selectively inhibit growth of Gram-negative *P. aeruginosa* by specific interactions of aminoguanidine units on the quantum carbon dots and lipopolysaccharide residues in the outer membrane of *P. aeruginosa* [84]. 

Quaternized carbon quantum dots prepared from dimethyl diallyl ammonium chloride and glucose as precursors had MIC values between 12.5 μg/mL and 25 μg/mL for Gram-positive *S. epidermidis*, *S. aureus*, MRSA and *E. faecalis*, ranging up to 50 μg/mL for Gram-negative *E. coli* and *P. aeruginosa* (also see Table 2) [90]. Proteomic analyses suggested that the quaternized carbon quantum dots acted on ribosomal proteins in Gram-positive bacteria and downregulated metabolization-related proteins of Gram-negative bacteria. Real-time quantitative PCR confirmed differences in expression level of genes related to these proteins in Gram-positive and Gram-negative strains.

### 4.6. Synergistic Use of Carbon Quantum Dots Combined with Antibiotics or Photosensitizers

Although carbon quantum dots exhibit antibacterial activities as a stand-alone antimicrobial, clinical translation and market introduction often proceeds stepwise. Cell wall damage inflicted by carbon quantum dots facilitates entry of antibiotics through pores created into a bacterium to enhance killing. Matrix disruption and reduction of volumetric bacterial densities in infectious biofilms by carbon quantum dots enhance antibiotic penetration and killing in a biofilm (Figure 9E) [75]. Additionally, photoactivated carbon quantum dots combined with commonly used photosensitizers, such as methylene blue and toluidine blue, achieve higher ROS generation than photosensitizers alone under visible light illumination, thus resulting in an enhanced, synergistic killing of bacteria [34,58]. This implies that combined use of carbon quantum dots with existing antibiotics might be a good first step in clinical translation.

### 4.7. Use of Carbon Quantum Dots in In Vivo Studies

In vivo studies are either performed with respect to evaluating antibacterial efficacies of carbon quantum dots or to establish their biosafety. Carbon is generally considered non-toxic, and quaternized carbon quantum dots, for instance, showed no obvious toxic side-effects during experimental treatment of infected wounds in rats [90] or pneumonia in mice [91]. Systematic evaluation of the biosafety of photoluminescent carbon quantum dots, synthesized through nitric acid oxidation, demonstrated no acute or sub-acute toxicity nor genotoxicity [101]. Additionally, no abnormalities or lesions were observed in the organs of mice. However, quantum dots intended for imaging purposes have sometimes been found to be less harmless, but these quantum dots are usually not carbon based, having a cadmium-telluride [102] or cadmium-selenium core [103]. Non-cytotoxicity of cadmium-selenide quantum dots could be enhanced by polyethylene glycol coating. 

In vivo evaluation of the antibacterial efficacy of carbon quantum dots, so far, has pointed out that quaternized carbon quantum synthesized from organic sources were equally efficacious in treating infection in rodents as antibiotics. The application of quaternized carbon quantum dots to wounds infected by a combination of *S. aureus* and *P. aeruginosa* in rats was equally efficacious as treatment with levofloxacin [90]. Additionally, positively charged carbon quantum dots prepared by heating of polyethene polyamine could be used to treat wounds infected by a combination of *S. aureus* and *E. coli*, with an efficacy equal to levofloxacin [92]. Nasally applied quaternized carbon quantum dots caused regression of MRSA-induced pneumonia in mice, with an efficacy similar to the one of vancomycin, by affecting protein translation, posttranslational modification and protein turnover in bacteria [91]. Collectively, we can conclude that quantum carbon dots are bio-safe, while quaternized carbon quantum dots appear promising for the treatment of infection, although their efficacy is not higher than that of antibiotics. Nevertheless, they may be useful, as they appear to be efficacious against infections by antibiotic-resistant strains.

## 5. Conclusions and Outlook

Carbon quantum dots can be effectively synthesized from both natural and synthetic carbon sources using various experimental methods. Among the synthetic carbon sources distinguished in this review, organic carbon sources more broadly cover the entire spectrum of antibacterial activities distinguished here than inorganic carbon sources. By maintaining critical, chemical functional groups of their source materials, carbon quantum dots can acquire desired properties for antibacterial applications towards both Gram-positive and Gram-negative bacterial strains. Carbon quantum dots derived through heating (pyrolysis, hydrothermal methods or smoking) of antibiotics and natural carbon sources, such as medicinal herbs and plants or probiotic bacteria, are ideal sources for the synthesis of antibacterial carbon quantum dots, since essential properties of carbon sources are inherited by carbon quantum dots. Quaternized carbon quantum dots have been found to be equally efficacious for controlling infections in rodents as antibiotics. Antibacterial activity of carbon quantum dots predominantly involves cell wall damage and disruption of the matrix of infectious biofilms (“dispersal”) through the generation of ROS. Synergistic antibacterial efficacy of carbon quantum dots, when combined with existing antibiotics and the added advantage of antibiotic-derived carbon quantum dots to yield a lower chance of inducing bacterial resistance than their source antibiotics, make carbon quantum dots attractive for further clinical translation and large-scale clinical use.

## Figures and Tables

**Table 1:** Summary of methods to synthesize carbon quantum dots, including suitable carbon sources, their advantages and disadvantages.

Method	Schematic Synthesis	Advantages	Disadvantages	Reference
Acidic oxidation	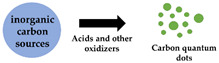	Large scale synthesis	Poor size control, risk of burning or explosion, mainly inorganic carbon sources	[21,24,25,26,27,28,29,30,31,32,33,34,35,36]
Pyrolysis	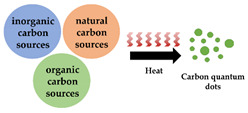	Avoids use of strong acids or alkalis, cost effective, suitable for widely different carbon sources	Poor size control	[19,37,38,39,40,41,42,43,44]
Hydrothermal synthesis	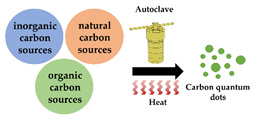	One step procedure, avoids use of strong acids or alkalis, cost effective, suitable for many carbon sources	Poor size control	[22,23,45,46,47,48,49,50,51,52,53]
Microwave-assisted synthesis	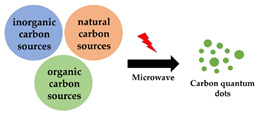	Short reaction time, suitable for many carbon sources	Poor size control	[21,54,55,56,57]
Laser irradiation	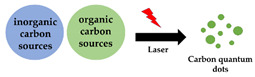	Short reaction time	Poor size control	[58,59,60]
Electrochemical synthesis	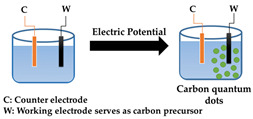	Good size control	Mainly inorganic sources, few available small molecule precursors	[12,61,62,63,64,65,66]
Nanoreactor-assisted synthesis	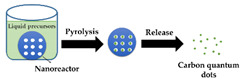	Good size control	Time consuming, nanoreactor preparation is difficult, only liquid precursors	[67,68,69,70]

**Table 2:** Summary of carbon quantum dots synthesized from different carbon sources, their antibacterial activity and efficacy (i.e., MIC values).

Carbon Source	Synthetic Method	Antibacterial Activity	Bacterial Strains Used	MIC * (µg/mL)	Ref.
**From Organic Reagents**					
Polyamine, polyamine combined with ammonium, dopamine	Pyrolysis, microwave-assisted synthesis	Bacterial killing through cell wall damage; ROS generation	Gram-positive *Staphylococcus aureus*, *Bacillus subtilis*, *Salmonella enterica*, methicillin-resistant *S. aureus* (MRSA)	0.9–8	[19,37,38]
			Gram-negative *Escherichia coli*, *Pseudomonas* *aeruginosa*	0.9–8	
Bis-quaternary ammonium salt	Hydrothermal method	Bacterial killing through cell wall damage; ROS generation; biofilm growth inhibition; biofilm dispersal through electrostatic interactions	Gram-positive MRSA, *S. aureus*	2–4	[47]
			Gram-negative *E. coli*, ampicillin-resistant *E. coli* (AREC)	8	
Dimethyloctadecyl- [3-(trimethoxysilyl)propyl]ammonium chloride	Hydrothermal method	Biofilm dispersal through electrostatic and hydrophobic interaction with Gram-positive bacteria	Gram-positive *S. aureus*	No MIC reported	[74]
			Gram-negative *E. coli*	No activity	
3-[2-(2- aminoethylamino)ethylamino]propyl-trimethoxysilane, glycerol, quaternary ammonium compound lauryl betaine	Pyrolysis	Bacterial killing through cell wall damage	Gram-positive *S. aureus*, *Micrococcus luteus*, *B. subtilis*	8 – 12	[89]
			Gram-negative *E. coli*, *P. aeruginosa*, *Proteusbacillus vulgaris*	>200	
Dimethyldiallyl ammonium chloride, glucose	Pyrolysis	Acted on ribosomal proteins in Gram-positive bacteria and downregulated metabolization-related proteins of Gram-negative bacteria	Gram-positive *S. aureus*, MRSA, *Staphylococcus epidermidis*, *Enterococcus faecalis*	12.5–25	[90]
			Gram-negative *E. coli*, *P. aeruginosa*	25–50	
Diallyldimethylammonium chloride, 2,3-epoxypropyltrimethylammonium chloride	Pyrolysis	Affected protein translation, posttranslational modification and protein turnover	Gram-positive *S. aureus*, MRSA, *S. epidermidis*, *Listera monocytogenes*, *E. faecalis*	5 – 20	[91]
			Gram-negative *E. coli*, *Serratia marcescens*, *Salmonella paratyphi-β*	No activity	
Citric acid, l-glutathion, polyethene polyamine	Pyrolysis	Bacterial killing through cell wall damage; ROS generation	Gram-positive *S. aureus*, MRSA, *L. monocytogenes*, *E. faecalis*	15–60	[92]
			Gram-negative *E. coli*, *P. aeruginosa*, *S. marcescens*, Drug-resistant *P. aeruginosa*, Drug-resistant *E. coli*	120–480	
Citric acid combined with aminoguanidine	Hydrothermal method	Bacterial killing through cell wall damage; biofilm growth inhibition	Gram-positive *S. aureus*, *B. cereus*	No activity	[84]
			Gram-negative *E. coli*, *Salmonella enteritidis*, *Salmonella typhimurium*, *P. aeruginosa*	0.5–1 (*P. aeruginosa*), >1000 (other strains)	
Citric acid combined with branched polyethyleneimine, 2,3-dimethylmaleic anhydride	Hydrothermal method	Biofilm dispersal through electrostatic and hydrophobic interaction with Gram-positive bacteria	Gram-positive *S. epidermidis*	No MIC reported	[75]
Gentamicin sulfate	Pyrolysis	Biofilm dispersal; bacterial killing through cell wall damage; ROS generation and maintenance of antibiotic features	Gram-positive *S. aureus*	0.002 (at pH 5.5)	[39]
			Gram-negative *E. coli*	0.203 (at pH 5.5)	
Ciprofloxacin hydrochloride	Hydrothermal method	Bacterial killing through maintenance of antibiotic features	Gram-positive *S. aureus*	1.0	[48]
			Gram-negative *E. coli*	0.025	
Metronidazole	Hydrothermal method	Bacterial killing through maintenance of antibiotic features	Gram-positive *S. mutans*	No activity	[49]
			Gram-negative *E. coli*, *Porphyromonas gingivalis*	No MIC reported	
Vitamin C	Electrochemical method	Bacterial killing through cell wall damage	Gram-positive *S. aureus*, *Bacillus* sp. *WL-6*, *B. Subtilis*	No MIC reported	[61]
			Gram-negative *E. coli*, AREC	No MIC reported	
Poly-oxyethylene, -oxypropylene, -oxyethylene Pluronic 68	Pyrolysis	Bacteria killing through ROS production upon blue light irradiation	Gram-positive *S. aureus*, *B. cereus*	No MIC reported	[76]
			Gram-negative *P. aeruginosa*	No MIC reported	
**From Inorganic Carbon Sources**					
Carbon nanopowder, 2,2′-(ethylenedioxy) bis(ethylamine)	Acidic oxidation	Bacterial killing through ROS production upon visible light irradiation	Gram-positive *B. subtilis*	64	[24,29,30,34,93]
			Gram-negative *E. coli*	64	
Graphite	Acidic oxidation	Bacterial killing through ROS generation under laser irradiation	Gram-positive MRSA, *S. aureus*	No MIC reported	[31,32,33]
			Gram-negative *E. coli*	No MIC reported	
Carbon fibers	Acidic oxidation	Biofilm dispersal through interference with the self-assembly of amyloid peptides	Gram-positive *S. aureus*	No MIC reported	[94]
**From Natural Carbon Sources**					
*Lactobacillus plantarum*	Hydrothermal methods	Biofilm growth inhibition	Gram-negative *E. coli*	No MIC reported	[23]
*Artemisia argyi* leaves	Smoking	Bacterial killing by cell wall damage through cell wall-related enzyme inhibition	Gram-positive *S. aureus*, *B. Subtilis*	No activity	[78]
			Gram-negative *E. coli*, *P. aeruginosa*, *P. vulgaris*	No MIC reported	
Cigarettes	Smoking	Bacterial killing through destruction of DNA double helix structure	Gram-positive *S. aureus*, AREC, *B. subtilis*	No MIC reported	[79]
			Gram-negative *E. coli*, kanamycin-resistant *E. coli*, *P. vulgaris*, *P. aeruginosa*	No MIC reported	

* MIC values for quantum carbon dots are based on weight per unit volume, but values may not be directly comparable with the traditional MIC value of antibiotics. MIC values of antibiotics refer to the weight of dissolved molecules, while carbon quantum dots are nanoparticles with diameters that are larger than of molecules.
